# Design and Interim Recruitment Outcomes of a Multi-Modal, Multi-Level Patient Navigation Intervention for Lung Cancer Screening in the Southeast U.S.

**DOI:** 10.3390/cancers17223633

**Published:** 2025-11-12

**Authors:** Marvella E. Ford, Louise Henderson, Alison Brenner, Vanessa B. Sheppard, Stephanie B. Wheeler, Tiffani Collins, Monique Williams, Rosuany Vélez Acevedo, Christopher Lyu, Chyanne Summers, Courtenay Scott, Aretha R. Polite-Powers, Sharvette J. Slaughter, Dana LaForte, Darin King, Amber S. McCoy, Jessica Zserai, Sherrick S. Hill, Melanie Slan, Steve Bradley-Bull, Neusolia Valmond, Angela M. Malek, Ellen Gomez, Megan R. Ellison, Robert A. Winn

**Affiliations:** 1 Department of Public Health Sciences, College of Medicine, Medical University of South Carolina, Charleston, SC 29425, USA; 2 Hollings Cancer Center, Medical University of South Carolina, Charleston, SC 29425, USA; scottc@musc.edu (C.S.); mccoyamb@musc.edu (A.S.M.); zserai@musc.edu (J.Z.); slan@musc.edu (M.S.); malek@musc.edu (A.M.M.); gomezel@musc.edu (E.G.); ellisome@musc.edu (M.R.E.); 3 Department of Biological & Physical Sciences, South Carolina State University, 300 College Street NE, Orangeburg, SC 29117, USA; 4 Lineberger Comprehensive Cancer Center (UNC), University of North Carolina at Chapel Hill, Chapel Hill, NC 27599, USA; louise_henderson@med.unc.edu (L.H.); alison.brenner@unc.edu (A.B.); stephanie_wheeler@med.unc.edu (S.B.W.); christopher_lyu@med.unc.edu (C.L.); chyanne_summers@med.unc.edu (C.S.); steve_bradley-bull@med.unc.edu (S.B.-B.); 5 Division of General Medicine and Clinical Epidemiology, College of Medicine, University of North Carolina at Chapel Hill, Chapel Hill, NC 27599, USA; 6 Virginia Commonwealth University Massey Comprehensive Cancer Center (VCU), Richmond, VA 23298, USA; vlbsheppard@vcu.edu (V.B.S.); collinslt@vcu.edu (T.C.); williamsm33@vcu.edu (M.W.); velezrn@vcu.edu (R.V.A.); hills3@vcu.edu (S.S.H.); valmondn@vcu.edu (N.V.); robert.winn@vcuhealth.org (R.A.W.); 7 Department of Social and Behavioral Sciences, School of Public Health, Virgina Commonwealth University, Richmond, VA 23284, USA; 8 Department of Health Policy and Management, Gillings School of Global Public Health, University of North Carolina at Chapel Hill, Chapel Hill, NC 27599, USA; 9 Fetter Health Care Network, Charleston, SC 29403, USA; aretha_jones@fetterhealthcare.org (A.R.P.-P.); drsharvette_slaughter@fetterhealthcare.org (S.J.S.); Dana_LaForte@fetterhealthcare.org (D.L.); darin_king@fetterhealthcare.org (D.K.); 10 Department of Internal Medicine, School of Medicine, Virgina Commonwealth University, Richmond, VA 23284, USA

**Keywords:** lung cancer screening, black patients, medically underserved, federally qualified health centers, patient navigation

## Abstract

**Background/Objectives:** Lung cancer is the leading cause of cancer death in the United States (U.S.). Virginia, South Carolina, and North Carolina are among the U.S. states with extraordinarily high rates of lung cancer mortality, particularly among Black residents. The current lung cancer screening guidelines, revised in 2021, support screening for younger, non-Medicare age-eligible individuals who smoke. However, their health insurance, if any, may not cover their screening. This lack of access could create more disparities in lung cancer mortality rates. **Methods:** To address this concern, the Virginia Commonwealth University Massey Comprehensive Cancer Center, the Medical University of South Carolina Hollings Cancer Center, and the University of North Carolina Chapel Hill Lineberger Comprehensive Cancer Center secured a four-year Stand Up To Cancer^®^ (SU2C) grant titled “Southeastern Consortium for Lung Cancer Screening (SC3) Study” with a novel aim to test the effectiveness of a multimodal, multilevel, barrier-focused patient navigation intervention to promote lung cancer screening among Black patients from federally qualified health centers. **Results:** A total of 170/675 Black participants have been recruited to date. The majority of participants (n = 134; 78.82%) were aged 55–74 years. Most participants were unmarried (n = 100; 58.82%), more than half had a high school education/GED or less (n = 111; 65.29%), most currently smoked (n = 142; 83.53%), and more males than females participated (n = 107; 62.94% male). Their reported lung cancer screening barriers, addressed by the patient navigators, were cost concerns, insurance coverage issues, and recent medical history precluding screening. **Conclusions with Relevance to Cancer Health Equity:** This SC3 study includes a unique lung cancer screening cohort that is in direct contrast to the predominantly White cohort in the National Lung Screening Trial. The SU2C study has created a novel, community-engaged approach to lung cancer screening navigation that could become the gold standard in high-risk medically underserved populations.

## Simple Summary

Lung cancer is the major cause of cancer death among people living in the United States (U.S.); death rates are highest among Black men. People as young as ages 50–64 who meet the U.S. lung cancer screening guidelines may receive screening, although they will typically not qualify for Medicare coverage for screening. Therefore, the study team obtained funding from Stand Up To Cancer^®^ (SU2C) to test a new patient navigation strategy to assist Black patients from federally qualified health centers with finding resources to overcome their lung cancer screening barriers. To date, 170/675 participants have been recruited. Most were unmarried (58.82%), had a high school education/GED or less (65.29%), currently smoked (83.53%), and were men (62.94%). Reported lung cancer screening barriers included cost and insurance issues. This innovative, community-engaged strategy to improve lung cancer screening rates and reduce lung cancer deaths in high-risk people could serve as a national model.

## 1. Introduction

### 1.1. Lung Cancer Mortality Rates in the United States (U.S.) and in Virginia (V.A.), South Carolina (S.C.), and North Carolina (N.C.)

Lung cancer is the leading cause of cancer death in the U.S. Lung cancer mortality rates are particularly high in southeastern U.S. states such as Virginia, South Carolina, and North Carolina [1]. In 2025, the lung cancer death rate per 100,000 population in Virginia is projected to be 58.1 for men and 41.9 for women; in South Carolina, these rates are expected to be 47.4 and 29.8, respectively; and in North Carolina, the rates are anticipated to be 46.8 and 30.7 [1]. These high lung cancer mortality rates, in comparison to the projected 2025 U.S. rates of 38.7 for men and 27.6 for women per 100,000 [1], are largely attributable to the social and contextual factors experienced by the residents of each state, such as high rates of poverty and unemployment, and lower levels of education, which translate into lack of access to preventive services such as lung cancer screening.

Lung cancer death rates are significantly higher for Black men than for members of any other racial/ethnic or sex group [2]. These higher death rates appear to be due to higher levels of lung cancer risk associated with social deprivation, which has been linked to lack of access to health care and poorer health outcomes. For example, in a recent study, Jafareian et al. [3] used latent class analysis to analyze data from a sample of 729 lung cancer patients who were seen at the University of Rochester from 2016 to 2017 [3]. The results showed that Black patients with lung cancer experienced higher levels of social deprivation than White patients (*p* < 0.05), and rural lung cancer patients experienced higher levels of social deprivation than urban patients (*p* < 0.05). Social deprivation was measured using the 5-year Social Deprivation Index, which measures the level of area-level social deprivation associated with lack of access to health care as well as health outcomes [4].

### 1.2. The Importance of Lung Cancer Screening in Reducing Lung Cancer Mortality Rates

In 2013, low-dose CT was recommended by the U.S. Preventive Services Task Force (USPSTF) as an evidence-based screening modality for high-risk individuals with a smoking history of 30 pack years who were 55–80 years of age [5]. In their seminal American Thoracic Society Official Statement, Rivera et al. [6] noted the precarious position in which high-risk, younger people with few financial resources would be placed if the screening age were lowered, and recommended patient navigation as a potential solution to overcoming the financial and other systemic barriers to care that face many high-risk people who smoke who are screening age-eligible but not eligible to have their screening costs covered by Medicare.

In March of 2021, USPSTF lowered the lower boundary of screening-eligible age to 50 years. Unfortunately, this placed a number of economically challenged individuals in a difficult position; they were age-eligible to receive lung cancer screening, but they lacked the resources to successfully navigate from their places of primary care to the nearest available lung cancer screening site.

### 1.3. Barriers to Lung Cancer Screening

Disparities among Black patients in receipt of lung cancer screening, lung cancer surgery, and other forms of treatment are likely due to several factors, including socioeconomic status, misperceptions and biases (on the part of both patients and providers), suboptimal communication in physician-patient interactions, and/or significant financial hardship associated with receiving care [7,8].

One in three Americans experiences significant financial hardship as a result of medical care, with the greatest burden in medically underserved populations, including Black and rural populations. Harmful, care-altering behavioral responses to the high cost of care, including lack of screening and delaying or foregoing medical treatment, may contribute to widely observed rural/non-rural and racial disparities in cancer mortality [9,10,11,12]. Because rural and Black individuals tend to have lower incomes, with fewer assets, higher unemployment, and higher rates of no or under-insurance than non-rural or White individuals, such out-of-pocket cost of care expenses constitute a greater share of monthly expenditures and thus create more burdensome material and psychological hardship. This hardship is most pronounced among rural communities with large minority populations [13]. Uninsured or “self-pay” individuals are generally responsible for paying the full cost of their care unless they can navigate the complex and uneven safety net of resources, including hospital charity care, entitlement programs, manufacturer assistance, and non-profit organizations’ aid, which are less available and harder to navigate in rural settings [14,15,16].

### 1.4. Patient Navigation

As lack of access to specialty care such as lung cancer screening is a healthcare disparity affecting Black people and rural populations in particular, multimodal, multilevel patient navigation interventions can potentially reduce multifaceted barriers to care and improve receipt of optimal screening and follow-up care [17,18]. As examples of barriers to care, many patients travel from their communities to more urban environments to receive lung cancer screening and follow-up treatment, which constitutes a significant economic burden, in terms of transportation, accommodation, lost wages, and opportunity costs of seeking care. A variety of financial assistance programs exist and provide much-needed financial assistance to such patients; however, these programs are fragmented, uncoordinated, and difficult to access, with cumbersome eligibility and paperwork requirements [15]. In addition, providers and support staff report that they lack both the time and knowledge to help patients navigate the increasingly complex and patchwork web of resources that do exist [15,19,20]. Therefore, a program led by trained navigators with a particular focus on addressing informational, social, and financial needs of program participants has the potential to overcome access barriers and promote more equitable screening, particularly for Black people, who are at high risk for poor cancer outcomes.

Patient navigation is an evidence-based approach that is designed to address the barriers to care that are outlined in the National Institute on Minority Health and Health Disparities (NIMHD) Research Framework [21]. Dr. Harold P. Freeman created one of the first patient navigation programs in 1990 to help women navigate the process of breast cancer screening and follow-up care [18,22]. Patient navigation is based on adult learning, social cognitive, and social support theory/competency evolution and is a barrier-focused intervention designed to ensure timely/efficient access to needed health services [23]. Navigators focus on case identification, addressing individual (fear, anxiety), organizational (screening locations/hours), economic, and sociocultural (mistrust of the health care system) barriers to care, and implementing a care plan [24,25]. Patient navigation interventions have been effective in navigating patients to cancer screening, through the diagnostic workup and resolution process, and through cancer treatment [17,19]. As lack of access to specialty care is a healthcare disparity affecting Black and rural populations, in particular, multimodal, multilevel patient navigation interventions can potentially reduce multifaceted barriers to care and improve receipt of optimal screening and follow-up care [23,26].

The objective of this paper was to describe the design and interim recruitment results of a four-year Southeastern Consortium for Lung Cancer Screening SC3 multimodal and multilevel patient navigation intervention that was developed to promote lung cancer screening among rural, urban, and medically underserved Black patients from federally qualified health centers. The intervention aims to reduce barriers to ensure timely access to screening.

## 2. Materials and Methods

### 2.1. Institutional Review Board Approval

Virginia Commonwealth University Massey Cancer Center served as the IRB of record for the study. The IRB protocol number is MCC SU2C-WINN.

### 2.2. Participant Inclusion Criteria

Eligible participants were required to meet the following criteria:

- Meets current USPSTF guidelines for lung cancer screening
- Adults aged 50 to 80 years
- 20 pack-year smoking history (Note: A pack-year is a way of calculating how much a person has smoked in their lifetime. One pack-year is the equivalent of smoking an average of 20 cigarettes—1 pack—per day for a year.)
- Currently smokes or has quit smoking within the past 15 years
- Identifies as Black or African American (Note: Both Hispanic/Latino and Non-Hispanic/Latino patients are eligible as long as they also identify as Black or African American [e.g.*,* Afro-Latino]).
- Willing to complete all navigation-related study activities
- Able to understand and the willingness to sign a written informed consent document

After receiving the referrals of potentially eligible EHR-defined Black participants from the federally qualified health centers, the SC3 study team members then administer the Baseline Survey, which serves as a means of confirming, based on the participants’ self-identification, their race/ethnicity.

### 2.3. Federally Qualified Health Center Partnerships

As Korn et al. [7] noted, promoting cancer screening in focal population groups that face barriers related to the social drivers of health, as outlined above, benefits from establishing multi-level partnerships with providers, health care settings, and patients [7]. These partnerships can be helpful in identifying patients who are most at risk of developing lung cancer and not receiving lung cancer screening, to link these patients with patient navigators who can help to resolve screening barriers and guide the patients to lung cancer screening sites near their communities. Federally qualified health centers (FQHCs) represent an ideal partner for lung cancer screening. These community-based organizations provide comprehensive primary care services to all patients, regardless of their ability to pay or their health insurance status [27]. FQHCs tend to serve racially and ethnically diverse patient populations. Their patients typically have high smoking prevalence. Two of the cancer center sites are employing an in-person, face-to-face recruitment and eConsent strategy with their FQHC partners, while the other cancer center is relying primarily on remote contact with potential participants to describe the study and to obtain consent from them.

**Virginia and Virginia Commonwealth University Massey Comprehensive Cancer Center FQHC Partner***:* The FQHC is a primary care, dental, and mental health center, and is the flagship clinic for a network of three FQHCs. Located in the predominantly Black East End community in Richmond, the FQHC serves approximately 12,469 patients per year, 69% of whom are Black, 61% are publicly insured, and 39% are ages 50+. As of 2018, the FQHC reported the second-lowest tobacco screening and cessation intervention (75% of eligible) among all FQHCs in the state.

**North Carolina and University of North Carolina-Chapel Hill Lineberger Comprehensive Cancer Center FQHC Partners***:* LCCC/UNC is partnering with two FQHCs in North Carolina. The first FQHC has 25 locations, serving 16 counties in western North Carolina that are all designated as medically underserved. In 2024, the FQHC served over 100,000 patients, including 25% Black, and 81% living at or below the federal poverty level (FPL), and 92% living below 200% FPL [28,29]. About 81% of the counties served by the first FQHC had higher lung cancer incidence rates than the state average (62.10 per 100,000), with the highest at 76.9 per 100,000 (Randolph County) [29]. Adult smoking in the FQHC is also higher than the state average (19% versus 17%) [29,30]. About 95% of adults seen by the FQHC have been screened for tobacco use and received smoking cessation counseling [3]. However, only 50% of eligible people who smoke in the FQHC have received lung cancer screening [3].

The second FQHC in North Carolina has 7 locations, serving 5 rural counties in eastern North Carolina that are all designated as medically underserved. In 2024, the FQHC served approximately 18,000 patients, including 52% Black, 74% living at or below the FPL, and 90% living 200% below the FPL. The lung cancer incidence in the FQHC serving area ranged from 46.9 (Gates) to 65.4 (Washington) per 100,000 [29]. Adult smoking in the FQHC is higher than the state average (19% versus 17%) [5,30]. About 76% of adults seen by the FQHC have been screened for tobacco use and received smoking cessation counseling [5]. About 61% of eligible people who smoke in the FQHC have received lung cancer screening [5].

**South Carolina and Medical University of South Carolina Hollings Cancer Center FQHC Partner:** The FQHC partner provides comprehensive services for insured, uninsured, and underserved residents in Berkeley, Charleston, Colleton, and Dorchester Counties. The FQHC network includes 20 different sites, many located along the I-95 Corridor. The network provides care for uninsured and underinsured people in medically underserved areas. Among the 15,489 patients served by the FQHC network, 2110 or 13.6% are tobacco users, and of those, 1289 (61%) are Black.

### 2.4. Centralized Patient Navigation Intervention

In the (SC3) study, centralized patient navigators were trained to help patients who were referred from each participating FQHC navigate the clinical, logistical, and financial aspects of lung cancer screening. The navigators employed case management approaches based on social work principles. The experience of patient navigation was tailored to the individual participant.

**Figure 1:**
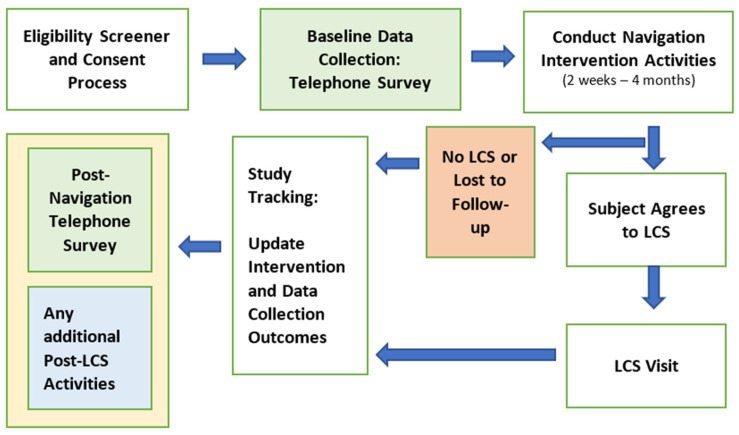
Schematic Flowchart of the Study Design.

The duration of the (SC3) study navigation intervention for patients was approximately 2 weeks to 4 months, depending on each individual patient’s needs. Patients were re-contacted by the navigator every 2 to 4 weeks until lung cancer screening was complete (or refusal of lung cancer screening had been documented) and post-intervention outcome measures were complete. After completing all LCS-related activities (refusal to screen, normal LCS results, or abnormal results with connection to thoracic oncology), patients completed the post-intervention survey (Figure 1).

Patient Navigation Training Modules. The navigators represented varied backgrounds, ranging from a licensed clinical social worker to a program coordinator. The main qualifications for the navigators’ role include:

- Superb interpersonal skills
- The ability to engage in active listening with the participants
- Problem-solving skills
- Knowledge of available resources
- Familiarity with the study’s research processes

In total, eight navigators were trained. The training sessions were led by the Principal Investigator and a Co-Investigator. All of the navigators participated in the training sessions at the same time. Each live, virtual training session lasted approximately 2 h.

The navigation training developed by the research team consisted of the following 10 modules (Table 1):

### 2.5. Conceptual Framework of the Patient Navigation Intervention

The SC3 Study navigation intervention addressed the following four domains of barriers to lung cancer screening [24,25]. Within this conceptual framework and in the context of the present study, individual barriers refer to perceived susceptibility to lung cancer; organizational barriers refer to systemic factors that limit access to lung cancer screening, such as difficulty scheduling a screening appointment; economic barriers refer to factors such as transportation issues; and sociocultural barriers refer to factors such as perceived social distance from lung cancer screening sites:

- Individual
- Organizational
- Economic
- Sociocultural

The SC3 centralized navigation approach was telephone-based, supplemented by texts. The navigation intervention did not include any in-person encounters. At the first contact, the navigators assessed the participant’s barriers in each of these domains. Any follow-up contacts, if necessary, were used to help the participant overcome these barriers to screening. Specific interventional navigation activities that were used are presented by domain, as shown in Table 2.

### 2.6. NCI Barrier Plan Form

**Figure 2:**
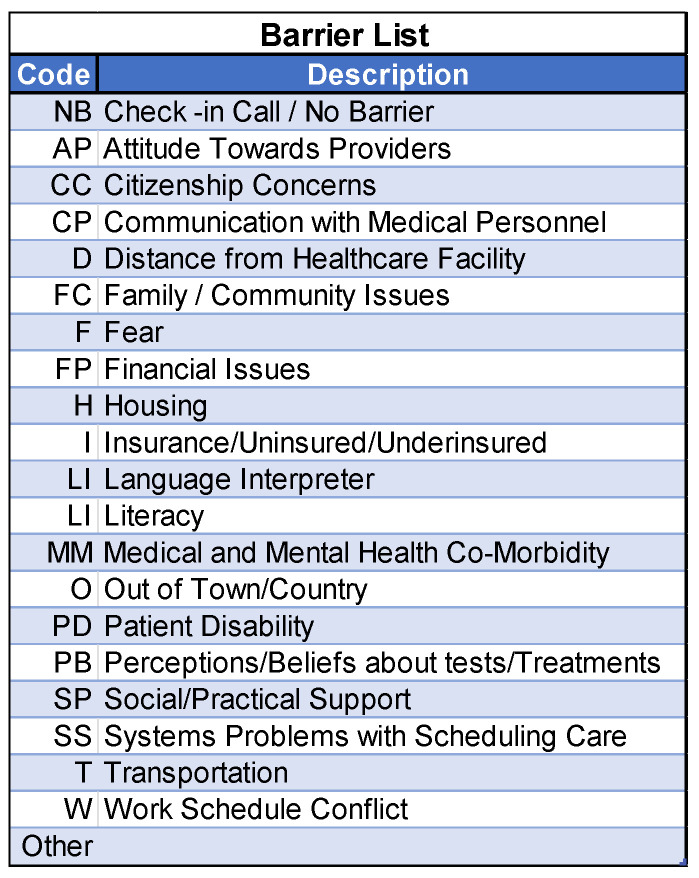
Examples of categories of barriers within the patient navigation intervention.

**Figure 3:**
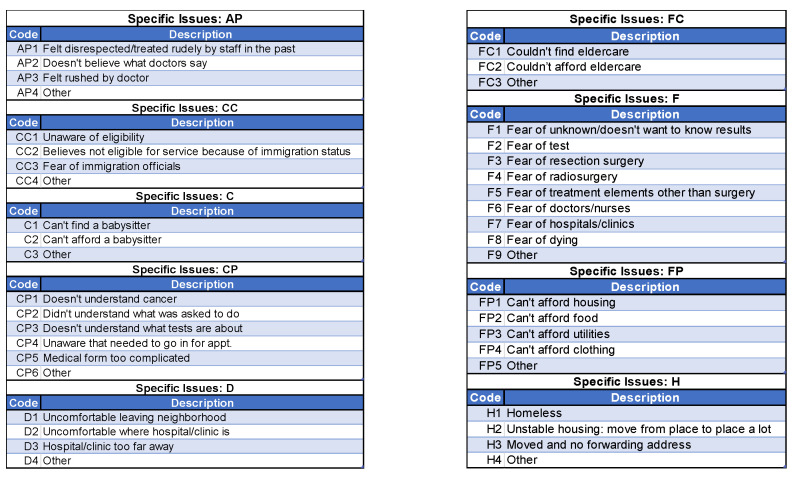
Examples of expanded categories of barriers within the patient navigation intervention.

**Figure 4:**
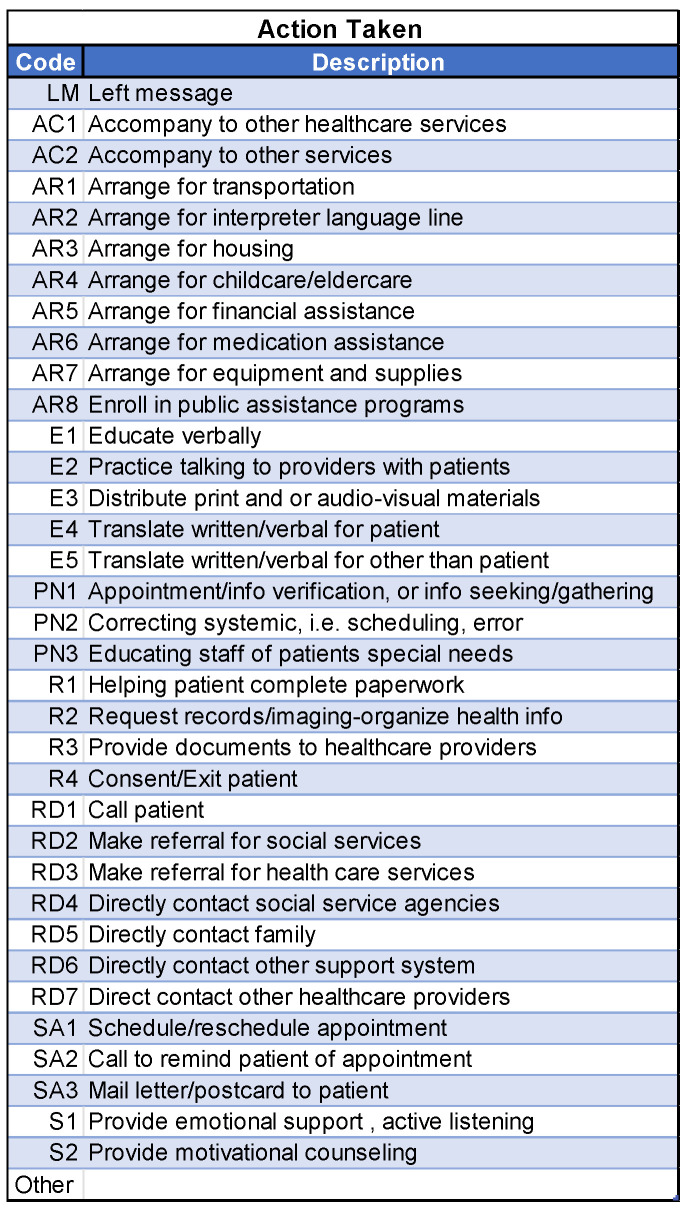
The general categories of actions taken by the study’s patient navigators to address the barriers identified by each participant.

The patient navigators actively asked about and addressed patients’ needs and barriers to care. To document each interaction with the participants, the navigators employed a web-based version of the NIH/NCI PN Barrier Checklist, used within the context of a Research Electronic Data Capture (REDCap) data management system. Study data were collected and managed using REDCap electronic data capture tools hosted at the Medical University of South Carolina (https://projectredcap.org/software/, accessed on 5 November 2025). The system elicits specific barriers that could prevent patients from receiving lung cancer screening. For each identified barrier, the REDCap system then uses branching logic to link to a barrier plan that the navigator will use to address and resolve the barrier. For example, if a patient stated that their initial barrier was related to communication with medical personnel (coded as CP in Figure 2), the navigator would then ask whether the communication was related to a lack of understanding of what cancer is, not understanding what the patient is being asked to do, not understanding what the medical tests mean or how they will be used, being unaware that they needed to schedule an appointment, feeling that the medical forms are too complicated, or another reason, coded as “Other” (Figure 3). The specific actions each navigator took to address and resolve the barriers presented by their assigned participants are shown in Figure 4, which shows drop-down boxes to capture the general categories of actions taken by the navigators, which are then linked to more detailed expanded fields to allow the navigators to describe each action in greater detail, including using text fields.

## 3. Results

To date, 170 Black participants have been enrolled in the SC3 study. The majority of participants (n = 134; 78.82%) were aged 55–74 years. Men comprised 107 (62.94%) of the participants. Only 26 (15.29%) of the participants were married or living with a partner. More than half (n= 111; 65.29%) of the participants completed high school/GED or had less than a high school education as their highest level of education. The majority of participants (n = 142; 83.53%) were people who currently smoked, and among those who quit, only 6 (3.53%) quit 10–15 years ago. The median pack years of the participants was 28 years.

As shown in Table 3, these SC3 study data are in direct contrast to the National Lung Screening Trial (NLST) data, which showed that 73.38% of the participants were aged 55–64 years; women comprised only 40.99% of participants, Black participants comprised only 4.48% of the total sample, 66.67% of participants were married or living with a partner, only 6.14% of participants had a high school/GED degree, and none had less than a high school education. Only 16.86% of participants in the NLST were people who currently smoke, and 19.67% had quit 10–15 years ago. The median pack years of the participants was 48.

The SC3 study participants’ comments related to study recruitment are shown in Table 4. As may be seen, the most common reasons for declining study participation were related to being fearful of the word “cancer,” not being interested in the study, being wary of the injury language in the informed consent document, feeling that the study was not a good fit, feeling that they were too busy to participate, having already made a decision to receive lung cancer screening, and focusing on other health problems.

The participants’ reported barriers to completing lung cancer screening were cost concerns, insurance coverage, and recent medical history that prevented study participation. These were the focal areas that were addressed by the study navigators.

## 4. Discussion

The purpose of this paper was to describe the design and interim recruitment results of a four-year Southeastern Consortium for Lung Cancer Screening (SC3) high-impact, multimodal, and multilevel patient navigation intervention to promote lung cancer screening among rural, urban, and medically underserved Black patients from federally qualified health centers. The intervention is focused on reducing barriers to ensure timely access to screening.

The SC3 study includes a unique lung cancer screening cohort that is younger, more likely to be unmarried, has lower levels of education, and includes more people who currently smoke than the predominantly White NLST cohort. Therefore, the study results will shed a significant amount of light on the effects of the patient navigation intervention in a population that is at high risk of developing lung cancer.

The SC3 study employs electronic informed consent (eConsent). This has proven to be a barrier to the potential participants for a number of reasons. First, the eConsent documents take up a significant amount of bandwidth, which is a problem for community members who may have limited funds to cover their monthly bandwidth charges. They have not been willing to use their data resources on the study documents. Additionally, many of the participants have shown a lack of knowledge and familiarity with using the eConsent process. Therefore, to overcome these barriers, two of the sites now employ in-person consenting using the eConsent process with a study iPad. To operationalize this process, the study recruitment team members travel to meet the potential participants at trusted sites that are near their homes, such as libraries, restaurants, workplaces, and community centers.

The investigators will evaluate their future navigation study findings in relation to those of similar studies. For example, Lee et al. [33] examined the effects of a telephone-based patient navigation intervention in enhancing lung cancer screening rates among patients in the context of a pragmatic randomized controlled trial in a safety-net health care system [33]. A total of 225 patients (55% of whom were Black, 15% of whom were Hispanic, and 30% of whom were White) were enrolled in the study navigation arm. Among the 225 navigation study participants, the navigators identified 559 barriers to screening during 806 telephone calls with the participants. The barriers included personal barriers (40% of the total number of barriers), followed by provider (30%) and practical barriers (17%). During the intervention, a decrease of 80% was seen in provider-related barriers (*p* = 0.008).

In a comparable study, Percac-Lima et al. [34] evaluated the effectiveness of a patient navigation intervention in increasing rates of receipt of lung cancer screening among 400 patients who were recruited from five community health centers that were affiliated with an academic medical center. These investigators found that among the 135 contacted and eligible patients, the navigators successfully reduced barriers to screening, and 124 (92%) of the participants received a chest CT. Thus, the intervention was found to be effective in increasing lung cancer screening in this group of high-risk people who smoked.

In another similar study, Bhalla et al. [35] evaluated data from 447 patients in a randomized trial of patient navigation to lung cancer screening (n = 225) or usual care (n = 222) in an urban safety-net hospital in Texas. The participants included a diverse group; 69% were racial/ethnic minorities. The investigators found that an organizational barrier, the lack of clinician ordering of the lung cancer screening, was a major impediment to receipt of screening among the navigated participants.

In future analyses, we will examine potential differences in the navigation results (including the barrier types, number of barriers, and resolution of barriers) of the current study, which includes a sample comprised entirely of Black patients, in comparison with the results from the more diverse samples in previous lung cancer screening navigation studies.

## 5. Conclusions

The SC3 study team has developed a novel strategy for lung cancer screening navigation that could become the gold standard of care for socially vulnerable and high-risk populations. The study participants, who were first-time recipients of lung cancer screening, likely only received it because of their participation in the study. With recent cuts to publicly funded health benefits, the need for a broader application of the patient navigation approach may be greater than ever. The investigators plan to complete a cost analysis as part of the final data assessment for a future outcomes paper. While the navigation intervention is resource-intensive, in comparison to the cost of treating late-stage lung cancer, it is anticipated that the navigation costs, which promote lung cancer screening and early detection, will be minimal.

## Figures and Tables

**Table 1:** SC3 Study Lung Cancer Screening Navigation Training Modules.

Module 1: Research 101	Objectives Describe the importance of research Describe five types of clinical studies/trials Identify steps involved in developing a study ▪ Discuss ethical violations in research that have: Helped with current ethical principles Shaped laws that regulate research about human subjects ▪ Describe the informed consent process ▪ Discuss best practices for administering informed consent
Module 1.2: Overview of Health Disparities in Lung Cancer Screening and Treatment	Objectives Define health disparities Provide evidence of health disparities in lung cancer screening and treatment Describe efforts to reduce these health disparities and describe anticipated outcomes of current efforts
Module 2: Patient Navigators: Roles and Responsibilities	Objectives Participate in the navigation training curriculum Become fluent in effective navigation skills Apply training to assist in recruitment & retention of Black patients to lung cancer screening Evaluate the training program and its impact on our ability to improve lung cancer screening rates
Module 3: Overcoming Barriers to Care	Objectives 1. Increase knowledge of social/cultural barriers ▪ Access to care issues ▪ Health literacy ▪ Insurance ▪ Access to medicine ▪ Structural access (navigation) 2. Expand knowledge of healthcare utilization factors ▪ Cost of healthcare ▪ Cultural beliefs ▪ Unconscious bias/discrimination
Module 4: Health Literacy	Objectives Demonstrate a basic understanding of key health literacy concepts Communicate the importance of health literacy to colleagues, grantees, and contractors Identify specific ways to integrate health literacy into our work
Module 5: Communication Exercise Tool	Objectives 1. Review the Communication Behavioral Style Questionnaire 2. Discuss the Interpretation Table ▪ Conscientious ▪ Direct ▪ Stabilizing ▪ Influencing 3. Describe how to communicate with different styles
Module 5.1: Effective Communication	Objective 1. Review and discuss: ▪ Barriers to communication ▪ Ways to overcome these barriers ▪ Communication tips and tricks ▪ Assertiveness ▪ Listening ▪ Being an informed navigator ▪ Ensuring patients understand ▪ Providing patient-based cross-cultural care ▪ Health literacy ▪ Patient communication skills 2. Cultural communication 3. Cultural Humility 4. Developing a system to help in the navigation process ▪ Plan-Do-Study-Act
Module 5.2: Communication and Patient Navigation	Objectives To provide coordinators with a self-assessment of their communication skills on which to build ideas for how to communicate in various situations To review collaborative communication and problem-solving
Module 6: Developing Cultural Competency/Cultural Humility in Cancer Clinical Trials Research	Objectives Understanding cultural humility ▪ Population Facts—Growing Diversity in the U.S. ▪ Why Cultural Competency/Humility Is Important to Cancer Clinical Trials Recruitment ▪ Defining Race, Ethnicity, and Culture ▪ Cultural Humility Activity #1 ▪ Cultural Humility Activity #2 ▪ Defining Cultural Humility ▪ Cultural Humility Activity #3 ▪ Cultural Humility Activity #4 ▪ Key Components of Culturally Humble Clinical Trials Recruitment ▪ Cultural Humility Activity #5
Module 6.1: Developing Cultural Humility in Health Care	Objectives 1. Understanding the Major Points Described in Crossing the Quality Chasm (Institute of Medicine 2002) [31] ▪ Six Aims for Improvement o Safety o Effectiveness o Patient-centeredness o Timeliness o Efficiency o Equity 2. Understanding population facts ▪ What is Race? o Genetics and Race ▪ Cancer Genetics and Race ▪ Race as a Social Construct Underlies Health Disparities o Ethnicity o Acculturation

**Table 2:** SC3 Study Lung Cancer Screening Navigational Activities by Domain.

**Individual**	**Economic**
Identifying each patient’s unique logistical and emotional needs and coordinating with other professional staff to develop effective solutions. Addressing the fear and mistrust that may be associated with lung cancer screening. Accompanying patients to their screening or treatment appointments as requested. Guiding patients through the lung cancer screening process. Providing support through active empathetic listening. Providing patients with sources of expert information about the recommended screening, including explaining the risks and benefits of lung cancer screening and assisting with decision making, if needed. Helping patients schedule and reschedule lung cancer screening and/or follow-up appointments. Providing appointment reminders and helping prepare patients for their appointments. Providing health education to patients about lung cancer risk, including smoking cessation counseling or referral to counseling. Detailed record-keeping on patient progress and outcomes.	Assessing patients’ individual financial situation and financial assistance goals, as well as collecting information about employment status, billing information, insurance status, and other indicators used to triage patients to the appropriate financial resource(s). Education and referral to financial resources such as local charity care, foundation-provided financial support, medication assistance programs, Medicaid, Medicare, private health insurance plans, Social Security Administration entitlements, and legal aid. Assistance with application submission and completion for financial aid resources Providing a checklist of resources they are potentially eligible for and a list of the personal paperwork (tax forms, W-2, pay stubs) needed to apply. Helping patients to understand their health insurance or to apply for health insurance, if needed. Linking patients in need to easy-to-access, centralized healthcare resources for the uninsured in their community, including through programs such as AccessHealth in South Carolina and NCCare360 in North Carolina.
**Organizational**	**Sociocultural**
Facilitating interaction and communication with healthcare staff and providers, including helping patients find language-concordant materials and resources. Assisting patients in finding ways to pay for their care by working with finance and billing staff at the healthcare site. Interacting with patients’ specialists to relay patients’ treatment concerns so that they can be addressed. Supporting patients as they navigate the complexities of the clinical environment Organizing and coordinating patient transportation services.	Acting as a resource to connect patients to community and social support services. Arranging for patients to hear testimonials from other patients with similar racial and ethnic backgrounds. Using lay language to describe the medical terms used by clinicians and explain patients’ concerns to their healthcare providers so that these concerns can be addressed.

**Table 3:** Characteristics of Current SC3 Study Cohort, as Compared to the NLST Cohort.

Characteristic	SC3 Study Cohort (N = 170)	NLST LDCT Arm [32] (N = 26,723)
**Age Group, years**		
50–54	7.65%	0.01%
55–64	44.12%	73.38%
65–74	34.70%	26.60%
75–79	8.82%	0.00%
Missing	4.71%	0.01%
**Sex**		
Female	37.06%	40.99%
Male	62.94%	59.01%
**Race**		
Black	100.00%	4.48%
**Marital status**		
Married/living with partner	15.29%	66.66%
Divorced	11.18%	19.44%
Separated	5.29%	1.26%
Widowed	11.76%	7.43%
Single	30.59%	4.70%
Don’t know/unsure	25.89%	0.51%
**Education**		
Less than high school	40.00%	6.14%
High school or GED	25.29%	23.48%
Post-high school training (No college)	0.00%	14.10%
Some College/Associate’s degree	18.82%	23.43%
Bachelor degree	5.89%	16.86%
Graduate School	0.00%	14.70%
Other	0.00%	0.85%
Don’t know/unsure/missing	10.00%	0.44%
**Smoking status**		
Current	83.53%	48.16%
Former	16.47%	51.84%
**Among those who quit, time (years) since quitting smoking**		
Within 4 years	6.47%	14.75%
4–9.9 years	4.11%	17.21%
10–15 years	3.53%	19.67%
Missing	2.36%	0.21%
**Median pack years**	28	48

Abbreviations: Southeastern Consortium for Lung Cancer Screening study = SC3; National Lung Screening Trial = NLST; Low-Dose CT = LDCT; General Educational Development High School Equivalency Diploma = GED.

**Table 4:** SC3 Study Participants’ Comments Related to Study Recruitment.

Common Reasons for Declining Participation	Common Concerns/Obstacles to Engaging in Lung Cancer Screening	Satisfaction with the Multimodal Navigation Approach
Hearing the word “cancer” Not interested in study participation or in getting screened Not comfortable enrolling in the study, citing the injury language in the informed consent form The study is not a good fit Too busy, no time, no interest in answering questions Already decided to get lung cancer screening Unwilling to go through the consent process Currently dealing with other health problems No reason provided—hung up	Cost concerns Insurance coverage issues Recent medical history that prevents receiving lung cancer screening	The majority of participants would recommend others to participate No changes to quality of life post-screening

## Data Availability

The data presented in this article will be made publicly available after analysis by the study investigators. Requests to access the data should be directed to Marvella E. Ford at fordmar@musc.edu.

## Abbreviations

SC3 Study	Southeastern Consortium for Lung Cancer Screening (SC3) Study
FQHC	Federally qualified health center
LCS	Lung cancer screening
SU2C	Stand Up To Cancer
USPSTF	U.S. Preventive Services Task Force
NIMHD	National Institute on Minority Health and Health Disparities
NLST	National Lung Screening Trial
